# Impedance Spectroscopy as a Methodology to Evaluate the Reactivity of Metakaolin Based Geopolymers

**DOI:** 10.3390/ma15238387

**Published:** 2022-11-25

**Authors:** Danilo Bordan Istuque, Alex Otávio Sanches, Marcelo Bortoletto, José Antônio Malmonge, Lourdes Soriano, María Victoria Borrachero, Jordi Payá, Mauro M. Tashima, Jorge Luis Akasaki

**Affiliations:** 1 MAC—Grupo de Pesquisa em Materiais Alternativos de Construção, Universidade Estadual Paulista (UNESP), Campus de Ilha Solteira, Av. Brasil Sul, 56-Centro, Ilha Solteira 15385-000, SP, Brazil; 2 Institute of Concrete Science and Technology (ICITECH), Universitat Politècnica de València, 46022 València, Spain

**Keywords:** geopolymer, metakaolin, impedance spectroscopy

## Abstract

The aim of this study was to use the electrical impedance spectroscopy technique (IS) to carry out a systematic study on the mechanism of metakaolin geopolymerization for up to 7 curing days. The study was developed on two batches of metakaolin (MK), and their reaction processes were compared. Interpretative fundamental elements were developed based on the effective electrical conductivity curves regarding the metakaolin geopolymerization. X-ray diffraction (XRD), Fourier transform infrared spectroscopy (FTIR) and scanning electron microscopy (SEM) were previously carried out and used to interpret and validate the electrical behavior of the fresh and hardened MK-based geopolymer pastes. The results highlighted the sensibility of the impedance technique to the identification and description of the MK geopolymerization process, as well as the changes resulting from even slight variations in the metakaolin composition. Furthermore, this indicated that the geopolymerization process in highly alkaline solutions could be divided into seven stages, including the processes of dissolution, nucleation, precipitation and formation of the gel and, eventually, the retraction/microcracks constitution. Late dissolution processes could be observed during the more advanced stages and were attributed to particles not being fully hydrated.

## 1. Introduction

Geopolymers are alternative binders produced by a chemical reaction between an aluminosilicate material and a highly alkaline activating solution [1]. A three-dimensional (3D) and amorphous structure based on [SiO_4_]^4−^ and [AlO_4_]^5−^ linked by oxygen atoms and balanced by the presence of alkali cations (Na^+^ or K^+^) is the primary product of such a type of reaction [2]. Zeolite structures (semi-crystalline structures) are also formed depending on the curing conditions, the number of water molecules and the type and concentration of the alkaline activating solution [2,3]. Several aluminosilicate materials (precursors) have been evaluated in the production of geopolymers, e.g., class-F fly ash, mining wastes, and metakaolin [4].

Metakaolin is a highly disordered (amorphous) aluminosilicate material originating from the kaolin clay calcination, where the dehydroxylation of kaolinite (Al_4_[Si_4_O_10_](OH)_8_) occurs in the range of 550 to 800 °C [5,6,7]. Kaolinite mineral is formed by two sheets of aluminate and silicate groups (1:1 structure) where adjacent layers are linked by hydrogen bonding of aluminol and siloxane groups [8]. The kaolinite dehydroxylation occurs by a gradual loss of structural water along with the changing of six-coordination aluminum to four-coordination one [7]. Due to its highly reactive behavior, metakaolin is largely used in studies related to the geopolymerization process [9].

The geopolymerization process involves the dissolution of solid alumino-silicate oxides in an alkaline solution in a generic way, the diffusion or transport of Al and Si complex ions dissolved from the particle surface to the interparticle space, and the formation of a gel phase resulting from polymerization between the added alkaline solution and Al^3+^ and Si^4+^ complexes. Due to the charge deficiency of Al^3+^ with respect to Si^4+^, Na^+^ or K^+^ cations are necessary to balance the presence of the Al^3+^ in the gel phase structure [10,11]. Water plays an important role in the entire process, as it is consumed during the dissolution of the precursors and eventually released during the geopolymerization process [3,12].

Although knowledge of the geopolymerization process has progressed, refined details regarding the reaction mechanisms, curing and hardening evolution have been poorly explained and need to be explored further [4,5,7,12,13]. In an attempt to assess such processes, various microstructural techniques, such as XRD, nuclear magnetic resonance (NMR), FTIR, Raman spectroscopy (RS) and SEM, have been applied [3]. Furthermore, calorimetric techniques (such as isothermal calorimetry) offer important results in distinguishing the geopolymerization processes due to exothermic reactions [14,15]. Few electrical measurements were applied to evaluate geopolymeric binders and have been reported in the literature, primarily based on impedance spectroscopy [16,17,18,19,20,21,22,23,24]. In the last decade, impedance spectroscopy has been successfully applied as a supplement to calorimetric techniques in the characterization of the hydration of Portland cement [25,26,27]. However, it is somewhat used to evaluate the geopolymerization process, given the complexity and overlapping of the physical/chemical reactions associated with ionic diffusion and water release processes [19]. The sum of these factors gives rise to difficulties in the individualization and interpretation of the geopolymer hydration processes in impedance spectroscopy measurements.

Despite the little attention received by such a technique in the literature, it has proved to be an important tool in the study of hydration processes at a micro and macro scale involving ionic release and transport processes, as well as in the microstructure formation and bulk conductivity. McCarter et al. [20] used the IS technique to monitor the hydration process in alkali-activated slag in the first 24–48 h of the reaction. The effects that change in the type of activating solution and its concentration exert on impedance curves were studied and analyzed through the conductance and capacitance formalism in the frequency range of 1–100 kHz. As a result, they found that such curves could be subdivided into a series of distinct regions which, depending on the type and concentration of the activator, could be considered as being indicative of the hydration stages, the chemical reaction rate and matrix stiffness changes.

In turn, Provis et al. [19] used the IS technique to monitor the in situ kinetics of geopolymer formation by alkali silicate activation of metakaolin at 40 °C. They also studied the action of various salts in the geopolymerization kinetic process and gel structure. Very similar curves to McCarter’s [20] work were obtained for the pure geopolymer and analyzed through conductivity formalism. The authors found a significant correlation between the conductivity and resistivity curves as a function of time to existing kinetic models. Through the technique, they were able to observe the influence that each type of salt had on the reaction kinetics and development of the geopolymer microstructure, confirming its high sensitivity.

Bing-hui Mo et al. [18] studied the influence of different curing temperatures on electrical conductivity obtained in situ for metakaolin-based geopolymers. They employed a mixture of sodium silicate and sodium hydroxide as an activating solution. The increase in the curing temperature showed a great influence on the conductivity curves, being correlated with the acceleration of the dissolution and polymerization processes. The results were compared with Vicat hardness measures and found to be highly consistent with them.

Zeng and Wang [17] studied the mechanical and electrical behavior of geopolymers obtained from different sources of fly ash. They observed that for early curing ages at a frequency of 10 Hz, the magnitude of the electrical impedance and the electrical resistance of the geopolymers tends to increase with curing time and are mainly controlled by the reaction rate. On the other hand, for more advanced curing periods, the electrical resistance of the geopolymers showed a great dependence on the composition. For one year after the initial reaction process, measurements indicated an increase in the impedance arcs and bulk resistance for all samples, suggesting that the geopolymerization process may extend over a long period.

Cui et al. [28] developed a systematic study of the dielectric properties of a new geopolymer synthesized from chemosynthetic Al_2_O_3_–2SiO_2_ powders and phosphoric acid. The study demonstrated that the dielectric properties of some geopolymers depended on the amount of free water and that the dielectric loss of geopolymers could not be reduced to a certain level as a consequence of the transfer of free sodium ions on the influence of the electrical field. On the other hand, the dielectric properties of the geopolymers, synthesized from phosphoric acid, showed dielectric losses of the order of 10^−3^ because of the reduction in the interdependence of ion transfer which, for such formulations, was considered negligible, as well as by decreasing or eliminating the free water.

Although several advances, such as those presented, have occurred in recent years, with the aim of applying the IS technique and understanding the impedance curves in their most diverse formalisms for the study of geopolymerization processes, many points still need to be clarified. In the literature, there is a large gap regarding in situ studies in more advanced periods of curing that exceed 24–48 h. In the great majority of the published reports, the process of geopolymerization is seen as well-defined, and they discuss the reaction of geopolymerization in well-identified stages. Thus, the role of possible non-hydrated phases and the effects of the overlap of these stages on the diffusion and reaction rate observed in the impedance curves are not considered. The studies do not directly discuss the role of water in more advanced curing periods (over 24 h) from in situ measurements.

In this way, this paper aims to study the geopolymerization process of MK-based material through impedance spectroscopy technique for up to 7 curing days. We intend to promote a better understanding of the physicochemical processes established during the reaction process, which are still poorly understood but dictate the final properties of the material. This paper presents a systematic study of the geopolymerization mechanism based on the effective electrical conductivity (σ_eff_) formalism. In particular, the study complements others [16,17,18,19,20], introducing basic elements for the understanding of σ_eff_ curves applied to the geopolymerization process. In the same way, we seek to understand the possible effects of the overlapping of geopolymerization processes caused by highly alkaline solutions in the impedance curves. Furthermore, we propose to understand the role of the processes of late reaction and the development of microstructure in the long term. Together with the IS technique, geopolymers and their precursors were characterized by XRD, TG, FTIR and SEM to support the statements made in the conductivity curves and better describe the process of geopolymerization and its effects on different batches of metakaolin.

## 2. Materials and Methods

### 2.1. Materials

In order to evaluate how even a slight difference in the chemical composition of the MK could affect the IS curves, measurements of two batches of MK (MK1 and MK2), supplied by Metacaulim do Brasil, were used to prepare the geopolymers. The chemical compositions of both precursors, obtained by means of X-ray fluorescence (XRF), are presented in Table 1.

**Figure 1:**
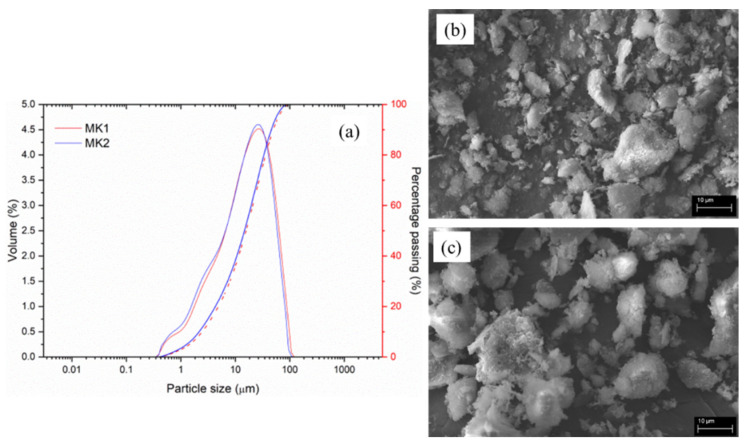
(**a**) Particle size distribution of MK1 and MK2. SEM micrographs of (**b**) MK1 and (**c**) MK2. Dark blue line and red dashed line represents the cummulative percentage (in mass) of both MKs.

Figure 1a shows the particle size distribution (%, in volume) for both MKs (determined using a Mastersizer 2000 analyzer from Malvern Instruments, Malvern, UK), and the micrographs (Figure 1b,c) demonstrate the small size of this type of material (EVO LS15—Zeiss, Oberkochen, Germany). Table 2 summarizes the granulometric parameters for both MKs.

**Figure 2:**
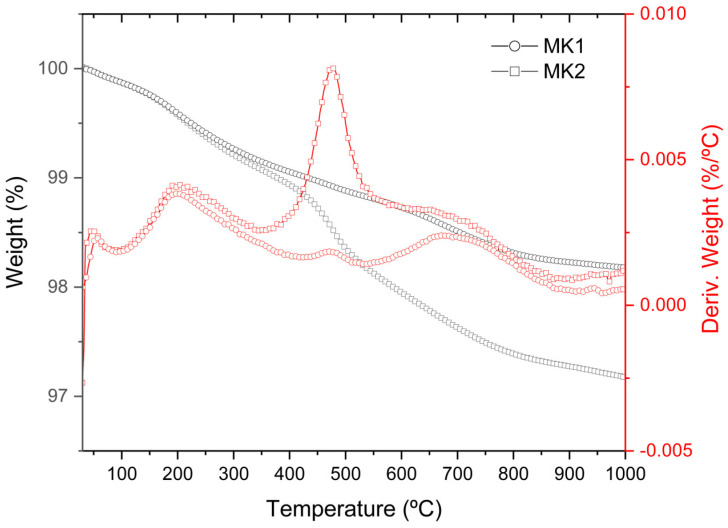
TG/DTG curves for samples MK1 and MK2.

Figure 2 illustrates the thermogravimetric curves (TG/DTG) of MK1 and MK2 samples. In particular, the transition process located at 380–580 °C is related to the dehydroxylation of residual kaolinite [29]. Thus, it can be noted that, for such a temperature range, the mass loss was approximately 0.2% for MK1 and 1.0% for the MK2 sample, showing a greater amount of remaining kaolinite in the MK2 sample.

NaOH pellets (98% purity) and Na_2_SiO_3_ (8.9% Na_2_O, 29.7% SiO_2_ and 61.4% H_2_O) were used to prepare the activating solutions and were supplied by Dinâmica Química™ (Indaiatuba, Brazil) and Diatom Química do Brasil Ltda.™ (Mogi das Cruzes, Brazil), respectively.

### 2.2. Experimental Procedure

#### 2.2.1. Geopolymer Production

The mix proportion of the geopolymeric pastes was fixed according to previous work [30], which reported better mechanical performance for geopolymers prepared with a concentration of NaOH of 8 M, SiO_2_/Na_2_O molar ratio of 1.6 and water/MK ratio of 0.6. After 1 h that the activating solutions were prepared (time required for the solutions to cool down to room temperature; 27 °C), MK was mixed with the activating solutions using mechanical stirring for 2 min, producing MK-geopolymer pastes. Such a mixing time was set to attend to the impedance measurements, which was time enough to obtain a homogeneous paste.

#### 2.2.2. Tests Performed

For the characterization of geopolymeric pastes, the geopolymerization reaction of the samples was stopped by the solvent-changing method. For this, the samples were immersed in acetone for 15 min and dried in an oven at a temperature of 60 °C for 1 h. After this process, a fraction of the sample was ground in a mortar until a mean particle size diameter under 125 µm was obtained and stored in Eppendorf for further utilization. In the same way, the precursors were subjected to an oven drying process (60 °C for 24 h) before any characterization. The morphology of the fractured surfaces was obtained using a scanning electron microscope (EVO LS15—Zeiss). The samples were placed in aluminum stubs with conductive carbon tape and metalized with gold. X-ray diffraction was performed for both precursors and pastes in different periods of geopolymerization (up to 7 curing days). The measurements were performed using Shimadzu XRD-6000 equipment with Kα radiation (λ = 1.5418 Å) in an angular range of 2Ɵ = 5° to 60° using a scan rate of 1°/min. The compound’s characterization was also studied using Fourier transform infrared spectroscopy. The pulverized samples were mixed in anhydrous potassium bromide in the proportion of 1:200 mg. The spectra were obtained using a NEXUS 670 spectrometer (from Nicolet Instrument Corporation, Madison, WI, USA) in the range of 4000 to 400 cm^−1^ (at 4 cm^−1^ resolution utilizing 128 scans). Thermogravimetric (TG/DTG) measurements were carried out in the temperature range 30 °C to 1000 °C, at a heating rate of 10 °C/min in a nitrogen atmosphere with a flow rate of 100 mL/min, using a Q600 model (from TA Instruments, New Castle, DE, USA). Platine sample pans were utilized, and approximately 30 mg was used for each sample.

**Figure 3:**
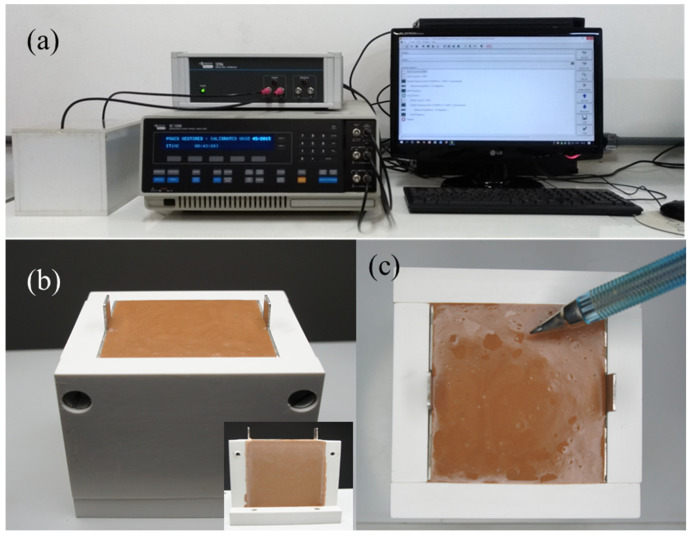
(**a**) Experimental equipment used to perform the impedance tests. (**b**) Image of the electrode systems. Inset: highlights the open mold on the side, illustrating the adhesion of the geopolymer to the electrodes. (**c**) Image of the surface of the MK sample with an emphasis on water expelled to the surface after 2 days.

In order to realize the impedance measurements (Figure 3), two electrodes made of stainless steel (50 mm × 50 mm × 1 mm) were placed at the ends of the opposite sides of the PVC mold (50 mm × 50 mm × 51 mm). Fresh MK-geopolymer pastes were poured into a mold between stainless steel electrodes after 2 min of mixing (Figure 3b). The molds were softly vibrated for 2 min to remove air bubbles. The molds were placed in a closed chamber in which the humidity had been stabilized at 95 ± 5%. Alligator-type connectors were placed on the electrodes and connected to the impedance apparatus (Solartron Impedance Analyzer with the 1296 Dielectric Interface, Leicester, UK). The chamber was then sealed to prevent any humidity variations. The humidity chamber was maintained in an air-conditioned room, where the temperature was 27 ± 2 °C. The measurements were collected in the frequency range from 1 Hz to 1 MHz for 7 days. The applied voltage was 1 V. The pre-determined measurement intervals were every 3 min for the first 1.5 h, every 10 min until 24 h of reaction and then every 1 h up to 7 days. Quantitative analyses were performed using the effective conductivity formalism σ_eff_ (Equation (1)) [25]:

(1) $$\sigma_{\mathrm{eff}}=\frac{L}{R_{b}A}$$

where L (m) and A (m^2^) are the length and effective area of the sample, and R_b_ (Ω) is the bulk resistivity.

## 3. Results

### 3.1. X-ray Diffractometry

**Figure 4:**
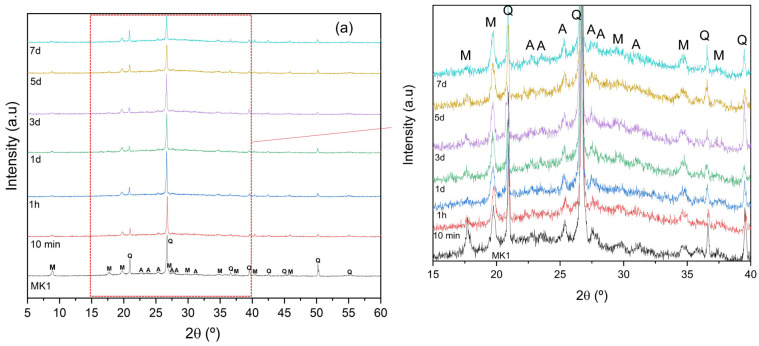

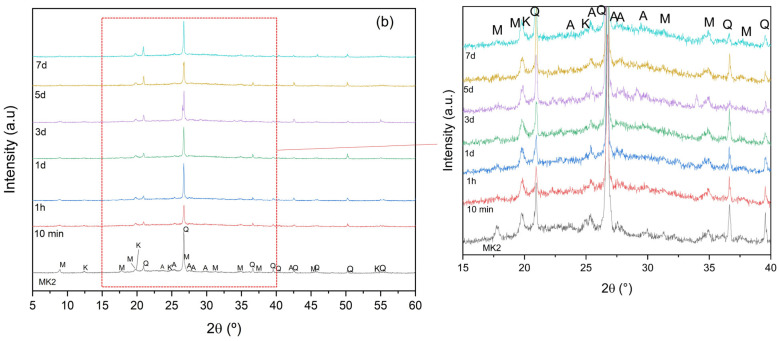
X-ray diffraction patterns for (**a**) MK1 and (**b**) MK2 and for the respective MK pastes as a function of geopolymerization time. (Key: Q—quartz (SiO_2_); M—muscovite mica (KAl_3_Si_3_O_10_(OH)_2_); A—albite (NaAlSi_3_O_8_); K—kaolinite (Al_2_Si_2_O_5_(OH)_4_).

Figure 4 illustrates the mineralogical analysis of both MK precursors, as well as the MK pastes for different curing times. Both MK precursors showed a diffuse halo located at 2θ = 18–38°, which is characteristic of the presence of amorphous phases [30,31,32]. As illustrated, the major crystalline phase is correlated to quartz in both cases (SiO_2_, PDFcard# 331161), although phases such as muscovite mica (KAl_3_Si_3_O_10_(OH)_2_ PDFcard# 210993) and albite (NaAlSi_3_O_8_, PDFcard#200554) were also observed, which agrees with the commonly reported literature [33,34,35]. The presence of kaolinite (Al_2_Si_2_O_5_(OH)_4_, PDFcard # 291488) could be observed only for the MK2 sample using this technique.

Compared to the precursors, the MK pastes presented an amorphous halo; however, this one presented a shift to greater angles (2θ = 16–40°), a direct consequence of the geopolymerization reaction [36]. In the same way, MK pastes, regardless of the geopolymerization period, illustrated the presence of crystalline phases such as quartz, muscovite mica, albite and (for the MK2 sample) kaolinite, indicating that none or only small amounts of these minerals reacted during the process [37,38,39,40]. In fact, the diffractograms indicate the existence of a partial dissolution of the phases, such as muscovite mica and albite, in both samples, as well as kaolinite for the MK2 sample with the evolution of the curing time [30,39,40].

### 3.2. Infrared Spectroscopy

**Figure 5:**
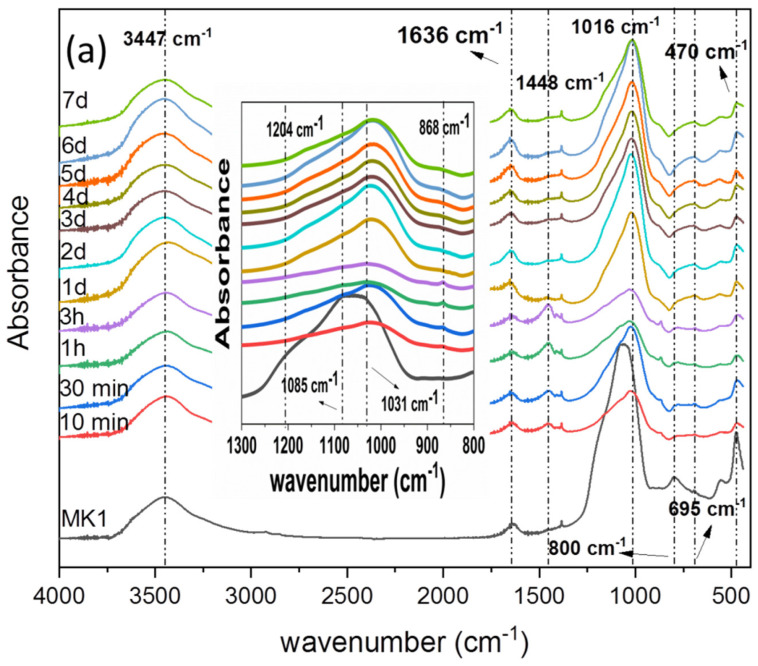

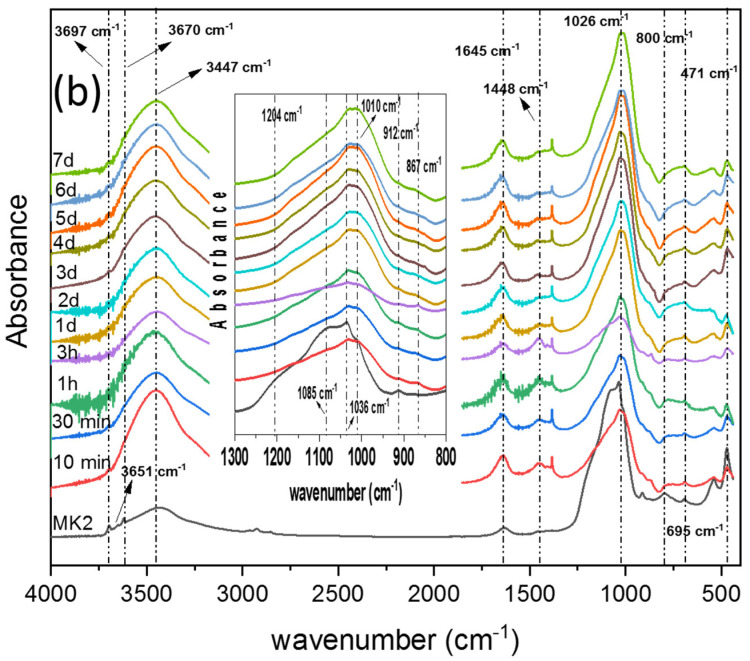
Infrared absorption spectra for samples (**a**) MK1 and (**b**) MK2, as a function of geopolymerization reaction. The main absorption bands are highlighted. Insets: magnification of the region between 1300 and 900 cm^−1^.

The infrared spectra of the MK1 and MK2 samples, as well as of the MK pastes as a function of the time reaction, are illustrated in Figure 5.

The broad band that appears in all spectra of the pastes, as well as in the MK1 and MK2 precursors, centered between 3434 and 3448 cm^−1^, together with the bands at 1385 and 1645 cm^−1^, are attributed to the stretching vibrations (-OH) and to bending (H-O-H) vibrations of the linked water molecules, respectively. These are adsorbed by the surface or trapped in the large cavities of the geopolymer [41,42,43].

The MK1 sample showed characteristic bands centered at 1085 and 1031 cm^−1^ and attributed to asymmetric stretching vibrations of Si-O-T bonds (T = Si or Al tetrahedron). The band at 470 cm^−1^ refers to the bending or stretching of the T-O-T bonds of aluminosilicates [30,41], while bands in the region of 521 to 550 cm^−1^ are related to the vibrational modes in the plane and in bending of the Si-O and Al-O bonds [30,44,45]. As observed in the spectra of the MK1 pastes, it is evident that the aforementioned bands tend to lose intensity as the geopolymerization time elapses. From 10 min to 3 h of reaction, the band centered at 800 cm^−1^ in the MK1 sample, assigned to the tetrahedral bending mode of T-O (T = Si or Al) and bending mode of the Si-O-Al bonds [29,43,46], loses a lot of its intensity and disappears after 3 h of curing. At the same time, the appearance of a band at about 695 cm^−1^ gradually increased in intensity up to 1 day. After that, this band suffered a significant enlargement and gradually increased in intensity, indicating the formation of Al (AlO_4_) tetrahedrons [42,47]. In the initial periods of curing, bands in the region of 700–790 cm^−1^ appear and can be attributed to Al-O vibrations bonds, corresponding to the spectrum of sodium aluminate [39]. This fact indicated the rupture of the Si-O-Al bonds in the first place, to the detriment of the Si-O-Si bonds, which can be justified by the lower bonding strength in the Al-O bonds compared to the Si-O bonds [39]. In turn, the large band observed in the range of 1300 to 900 cm^−1^, which appears in the early stages of geopolymerization, was attributed to the asymmetric stretching vibrations of the Si-O-T groups and is an indication of the extent of the poly-silanization reaction and the Al incorporation [35]. As the Al incorporation in the network tetrahedral positions increased, this band tended to shift to smaller wave numbers, which was centered on an average of 1025 cm^−1^ in 10 min and 1016 cm^−1^ in 7 days, as observed in the inset of Figure 5a. Such behavior is a consequence of lower binding forces between Al-O compared to Si-O bonds [48]. Another factor that can justify such displacement is found in the greater presence of non-binding oxygen atoms, which can mainly occur in the initial periods of geopolymerization. The band intensity at 1025 cm^−1^ has a general tendency to increase with the curing time, which is indicative of a stronger geopolymer framework. However, as can be seen in the Figure 5a inset, it is still possible to observe the presence of low-intensity bands at 1085 cm^−1^ and 1031 cm^−1^, which constitute the main band and, together with the bands at 470 cm^−1^ and 555 cm^−1^, corroborate the presence of unhydrated (or partially hydrated) particles during the entire geopolymerization process.

The band at 868 cm^−1^ is not characteristic of the MK spectrum; it appears at high intensity until 3 h, when it then loses intensity, suffering a small enlargement (see inset in Figure 5a). This was observed for all periods until 7 days. The observance of such a band, from the initial curing stages associated with its change in later curing days, suggests that the band at 868 cm^−1^ is due to stretching vibrations of the Si-O groups of the reaction products or unreacted activating solution in detriment of belonging carbonation [49,50].

In turn, the infrared spectra of the MK2 sample (Figure 5b) presented absorption bands with similar characteristics to those observed in the MK1 sample. In exception, we can mention the bands observed in 3696, 3651 and 3620 cm^−1^ attributed to the OH groups stretching vibrations, and the band in 912 cm^−1^ attributed to the bending type vibrations of the internal hydroxyl groups bonded to Al [29,30,41]. All of them belong to kaolinite. This fact indicates a greater presence of kaolinite in the MK2 chemical composition, to the detriment of the MK1 precursor, as shown by XRD and TG analysis.

The inset in Figure 5b clearly illustrates that the main band of the MK2 sample centered around 1050 cm^−1^ is composed of bands at 1085, 1036 and 1010 cm^−1^. Inobservance of the X-ray diffraction pattern of structures, such as pyrophyllite in the MK2 sample, suggests that the presence of such bands in the Figure 5b inset, for different curing periods, is due to the presence of MK particles not being fully hydrated [51].

Interestingly, in both samples, it was possible to observe a reduction in the intensity of the characteristic band of the geopolymer located between 1300 and 900 cm^−1^ after 1 h of geopolymerization; the reduction was at a maximum at 3 h when the general tendency of increase resumed. Similar behavior was observed by Król et al. [39] and attributed to the anionic mechanism that involves the formation of Si-O-Si bonds during the reaction between two oligomers followed by the elimination of a water molecule or even NaOH (depending on the terminal group).

### 3.3. Impedance Spectroscopy

**Figure 6:**
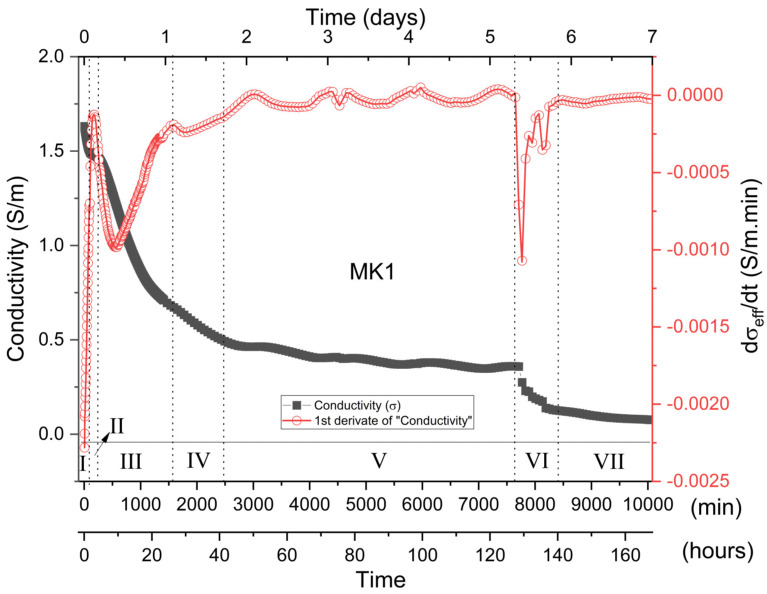
Effective electrical conductivity, σ_eff_, and its derivative, dσ_eff_/dt, as a function of the time reaction for MK1-based geopolymer. Highlighted regions of I–VII.

Figure 6 representatively illustrates the σ_eff_ behavior of the MK1 sample as a function of the time reaction, as well as its respective derivative (dσ_eff_/dt). It can clearly be seen that the σ_eff_ vs. t curve can be divided into seven distinct regions, characterizing different stages of the metakaolin geopolymerization process.

In the initial mixture between metakaolin and the alkaline sodium silicate solution (Na_2_SiO_3_), the alkaline attack of the metakaolin structures occurs with the consequent release of aluminate and silicate species in the solution. The large release of ions provided by the dissolution of metakaolin tends to promote a substantial increase in the solution conductivity values and is generally characterized in the literature by a peak. Some authors categorize this peak as the end of the dissolution process [19,20]. However, as can be seen in Figure 6, the high alkalinity of the mixture tended to promote a rapid initial dissolution so that the maximum of the curve is found in periods below 5 min when the measurements started. Similar behavior was observed by McCarter et al. [20] in a pioneering work, demonstrating the dependence of the behavior of the alkali-activated slag conductance as a function of the alkalinity of the solution. Preliminary measurements, carried out from shorter mixing periods, indicated that the maximum dissolution could be found in periods of less than 3 min, below the maximum measurement time for the scanning frequency range established in this study. In its physical–chemical interpretation, initial dissolution comprises the situation in which the dissolution rate is higher than the oligomer’s constitution (nucleation) and gel formation. This stage is very well defined in mixtures in which the alkalinity solution is not high [18,20]. In these, the nucleation process and subsequent precipitation are dependent on a slower dissolution process, which lasts for a longer period since many reactive species are required, as well as the establishment of diffusion processes for oligomer constitution and subsequent formation of the gel. However, in the case of highly alkaline solutions, the dissolution process is extremely fast [52], and, therefore, many reactive species are available for the constitution of chemical reactions. Thus, simultaneously with the dissolution process, the formation of oligomers is established, together with their precipitation and formation of the gel [14], therefore constituting what we classify as stage I.

**Figure 7:**
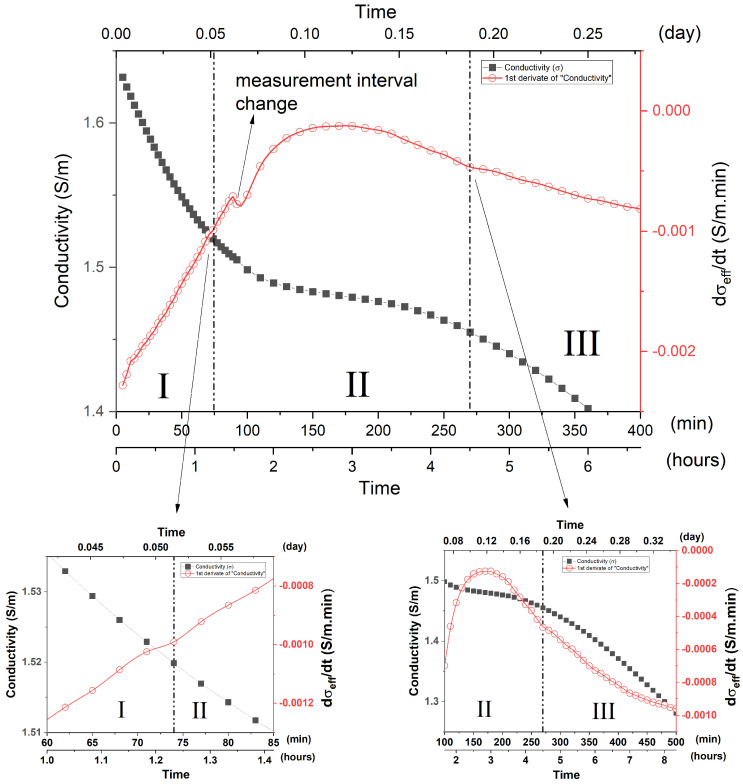
Effective electrical conductivity, σ_eff_, and its derivative, dσ_eff_/dt, as a function of the geopolymerization time, highlighting regions I and III: MK1 system. Insets illustrate the inflection points of the dσ_eff_/dt curve adopted as a delimiter between the referred stages.

**Stage I (up to 74 min)**—Stage I is characterized by a rapid reduction in σ_eff_ values. dσ_eff_/dt shows a negative value, but the curve shows a general upward trend (Figure 7). Such facts indicated that, although the dissolution process is still occurring in this period, the rate of oligomers constitution, precipitation and gel formation overlap the MK dissolution, therefore reducing σ_eff_ values.

The growing trend of the dσ_eff_/dt curve (less negative values) indicates that the rate at which σ_eff_ decreases over time is increasingly smaller, probably due to a greater ionic presence in the system from dissolution processes.

MK has a morphological structure characterized by large particles formed by stacked lamellae. The SEM images (Figure S1(A1–A3)) showed that, after 10 min of reaction, a large amount of geopolymer was formed. This partially encapsulated the particles that had not fully dissolved, altering the MK morphological structure. Particles not dissolved by the alkaline attack can be seen in the fracture as smooth angular-shaped structures. It can be seen in Figure S1(C1–C3) that, after 60 min of reaction, the morphological system structure tends to have a less compact, sponge-like morphology, consisting of globular units characteristic not only of the precipitation and growth of the geopolymer but also of the encapsulation process by the gel. However, it was possible to verify, throughout the whole period, the presence of undissolved or partially dissolved particles, probably composing quartz, kaolinite or MK, as seen in XRD, TG and infrared measurements. Given these observations, it is proposed that Stage I, in which the mixture solution is highly alkaline, comprises overlapping processes in which rapid dissolution is accompanied simultaneously by a rapid partial gel formation, as well as encapsulation of MK particles and other structures that are not fully dissolved. This event evidently originated in the catalytic effect promoted by the high alkalinity of the medium. The dissolution process is faster and more intense for the smallest MK particles, being slower for larger particles and occurring on the surface of MK [5,53]. The process of dissolution, nucleation and final synthesis of the gel occurs mainly on the surface of unhydrated MK particles, and it is dependent on diffusion processes [5]. Such a process becomes more interdependent with diffusion from the initial gel formation. Thus, in Stage I, partial encapsulation of the MK particles not fully dissolved occurs by the formed gel.

**Stage II (74 to 270 min)**—Stage II is characterized, in the σ_eff_ vs. t curve, by a plateau trend located in the range of 74 to 270 min. In this period, although σ_eff_ has a general reduction behavior, this reduction is smooth and tends to stabilize, although this does not occur directly, as represented by the derivative curve. Thus, the dσ_eff_/dt showed a negative value, and the curve trend showed an increase in behavior (Figure 7) until 180 min, which persists from Stage I. At 270 min, the dσ_eff_/dt curve subtly changes its inflection, thus characterizing the beginning of Stage III. Such associated facts are in line with the existence of a large ionic release, which overlaps the gel formation process, and may justify the intense bands observed up to 3 h in 868 cm^−1^ (Figure 5). Such ionic release may have originated from the late dissolution of larger MK particles or partially encapsulated ones, which require diffusion processes to be established. Anionic mechanisms contributed to such processes, as observed in infrared measurements, which can promote the release of water or even NaOH, which in their ionic state in solution, contribute to both the increase in dσ_eff_/dt and σ_eff_. The increase in the dσ_eff_/dt rate until 180 min indicates that the late dissolution system is intensified over any possible rate of gel formation, which may occur due to the intensification of diffusion processes and chemical attack from the surface of MK grains that are not fully hydrated. Such a process starts from Stage I and intensifies significantly in Stage II due to its temporal dependence. The SEM images (Figure S1(D1–E3)) illustrate the alteration of the morphological structure of the samples, in which the system takes on the globular shape originated by the chemical attack and deposition of the gel.

**Figure 8:**
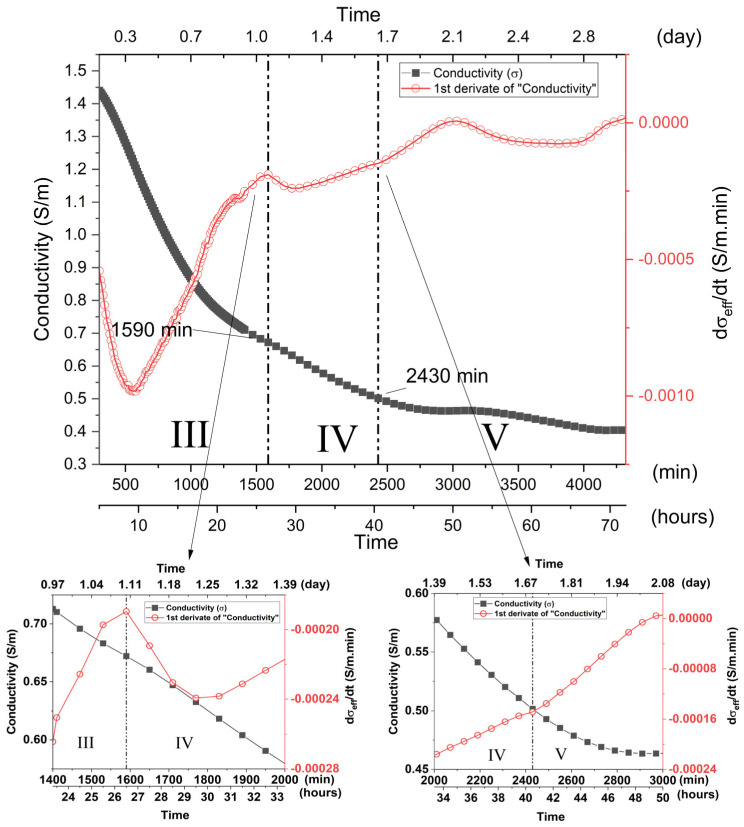
Electrical conductivity, σ_eff_, and its derivative, dσ_eff_/dt, as a function of the geopolymerization time, highlighting regions III–V. MK1 sample. Insets illustrate the inflection points of the dσ/dt curve adopted as a delimiter between the referred stages.

**Stage III (270 min to 1590 min)**—This stage is characterized by the continuous reduction in σ_eff_ and dσ_eff_/dt curve and reaches a local minimum of around 560 min (Figure 8). Such associations indicate a period of intense chemical activity in which there was the formation of a large amount of gel, thus reducing the ionic presence in the mixture and characterized by a substantial and gradual increase in the characteristic infrared band of the gel ranging from 1330 to 900 cm^−1^. The system tended to change its morphology again (Figure S2(A1–A3)), assuming a more compacted structure, characterizing an increase in the rigidity of the samples.

**Stage IV (1590 to 2430 min)**—Stage IV is characterized by a gradual reduction in the σ_eff_ as a function of the geopolymerization process (Figure 8). In such a period, there is a decrease in the dσ_eff_/dt rate, the curve presenting a local minimum of around 1770 min. It should be noted that this minimum illustrates a non-symmetrical profile, demonstrating an increasing trend after 1770 min and culminating in a local maximum in Stage V. Such characteristics indicated that different processes may be overlapping. It is assumed, in this way, that the initial reduction in σ_eff_ and dσ_eff_/dt must be associated with a predominant ion concentration reduction and consequent gel constitution. However, superimposed on such a reduction are the associated processes of late dissolution, which have a predominant effect after 1770 min; thus, dσ_eff_/dt increases until it reaches a maximum in Stage V (Figure 8). The SEM images illustrate a greater compaction of the samples due to the formation of a large amount of the geopolymeric gel. The presence of non-hydrated particles cannot be observed with such ease, although they are still present in the micrographs, as highlighted in Figure S2(B1–B3).

**Figure 9:**
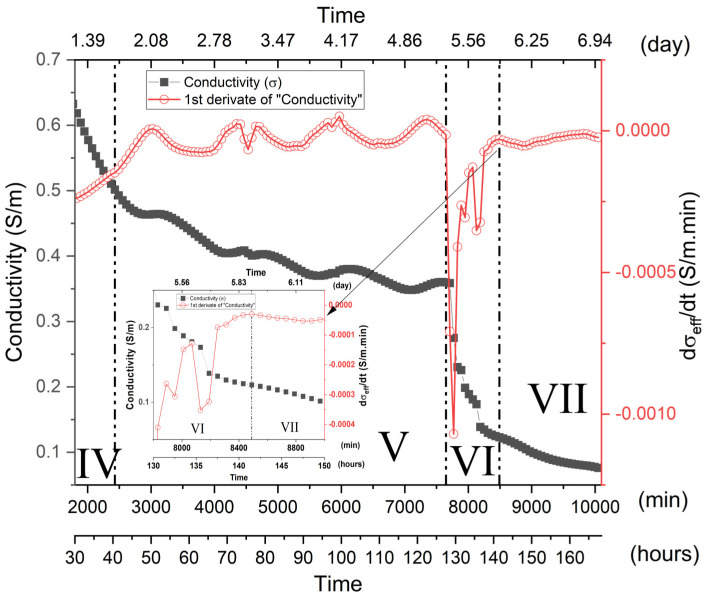
Effective electrical conductivity, σ_eff_, and its derivative, dσ_eff_/dt, as a function of the geopolymerization time, highlighting regions IV–VI (for MK1). Inset illustrates the inflection points of the dσ_eff_/dt curve adopted as a delimiter between stages VI and VII.

**Stage V (2430 min to 7650 min)**—Stage V is generally characterized by a softly trending reduction in the conductivity curve, indicating that most of the geopolymerization reaction has finished. Figure 9 shows that a series of local minimums and maximums can be seen, superimposed on the general reduction behavior, resulting in similar behavior in the dσ_eff_/dt curve. Such behavior indicates that, although a large part of the chemical activity related to the geopolymerization process has been concluded or reduced, late ion dissolution and release processes still persist, probably due to the alkaline attack of less reactive species and larger MK particles present in the mixture, as verified in infrared measurements and SEM images. The high magnification (5000×) SEM images (Figure S2(C3,D3,E3)) highlight and verify the presence of particles that are not fully hydrated. Throughout its entire surface, the presence of globular shaped structures attributed to the late gel formation can be observed. This fact corroborates the idea of a late chemical attack on the particle surfaces, dependent on diffusion processes. The same behavior was found for the later stages, as can be observed in the high magnification micrographs for Stages VI and VII (Figure S2(F3,G3)).

**Stage VI (7650 min to 8490 min)**—Stage VI is characterized by a drastic reduction in the electrical conductivity, with the dσ_eff_/dt curve presenting a sharp global minimum at 7770 min. Since the XRD and FTIR measurements do not indicate any structural alteration or formation of a new phase, it is believed that the sharp reduction in conductivity can be related to three distinct processes that can occur simultaneously and act to increase the contact resistance: (1) the first correlated to the microscopic detachment of the contact electrodes caused by the retraction processes of the geopolymer; (2) the second can be related to the formation of microcracks, that tend to generate a more resistive system by interrupting the continuity of the medium; (3) the third is related to a system depercolation process. Depercolation is characteristic of the geopolymerization process originated by the conjunct actions of reducing the geopolymerization process and water diffusion to the sample surface. As the geopolymerization reaction continues, water is released and fills the sample pores constituting a percolated structure. Throughout the measurement process, unbound water tends to diffuse to the sample surface (Figure 3c) with the reduction in the geopolymerization process; diffusion and evaporation processes contribute only to the system depercolation.

**Stage VII (8490 min)**—Stage VII is basically characterized by the stabilization tendency of σ_eff_ values. Figure S2(G1–G3) illustrate the SEM images after 7 days of curing. There was no significant morphological change compared to the previous stage, in which the samples presented a dense and compact structure characteristic of the gel.

#### Comparative Interpretation between Distinct Batches of Metakaolin

**Figure 10:**
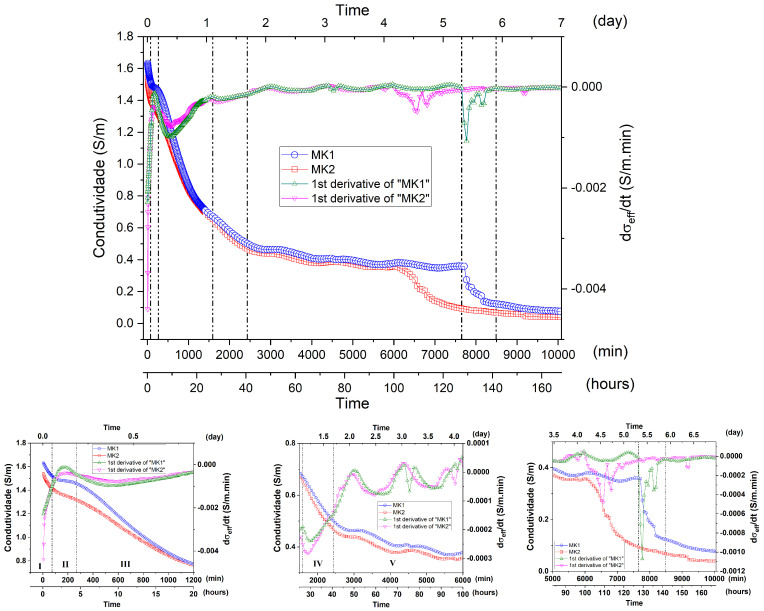
Electrical conductivity, σ, and its derivative, dσ/dt, as a function of the geopolymerization time for samples MK1 and MK2. Insets: comparative curves of the I–VII regions.

Figure 10 illustrates the comparative behavior of electrical conductivity as a function of time for two samples of metakaolin obtained from different batches (MK1 and MK2), as well as their respective derivative curves. The insets in Figure 10 are extensions referring to the different hydration stages. It was possible to notice, for the MK2 geopolymeric paste, the standard behavior of the σ_eff_ vs. t curve, detailed above for the geopolymerization process for the MK1 sample. It was observed that, for the initial measurement (Stage I), the electrical conductivity of the MK1 geopolymeric paste was slightly higher than that presented by MK2. This is due to greater chemical reactivity for the MK1 sample, represented by a greater initial dissolution process, in agreement with the lower amount of kaolinite present in the material, as demonstrated by XRD and TG measurements. The higher dissolution rate pointed to in the dσ_eff_/dt curve for the MK1 sample tends to counterbalance the σ_eff_ reduction with the simultaneous constitution of the gel, thus resulting in less conductivity decay compared to MK2. The broadest and most intense peak (dσ_eff_/dt curve) in Stage III for the MK1 sample indicates that there was greater variation in conductivity in the period, which reasonably reflects the greater initial dissolution of MK1 and greater constitution of the geopolymer in Stage III. After 800 min, the σ_eff_ values for both MK1 and MK2 geopolymeric pastes tend to be similar, indicating a reduction in the chemical activity for both. SEM micrographs (Figure S4(A1–A3)) obtained from the fractures of the MK2 sample referring to Stage III showed a greater amount of non-hydrated structures (comparative with MK1), with a small morphological alteration after 3 h of geopolymerization. In Stage IV, σ_eff_ of the samples starts to differ again, slightly higher for the MK1 sample, although there is no significant difference regarding dσ_eff_/dt. This fact extends throughout the respective Stage V of each sample. As discussed above, Stages IV and V are characterized by the reduction in chemical activity, as well as late geopolymerization processes, originating from particles that were not fully hydrated. The greater conductivity observed for the MK1 sample may be due to greater particle encapsulation coming from Stage III due to its higher conversion rate, which starts to suffer late hydration and is highly dependent on diffusion processes. Another factor that could contribute to such an event is related to possibly greater pore interconnection for the MK1 sample. Greater pore interconnection would facilitate the water expulsion release from the system and reduce the time for Stage VI. The greater presence of kaolinite in the MK2 sample (pointed out by XRD, FTIR and TG analyses) could be correlated with the greater σ_eff_ variation in Stage III for the MK1 sample, indicating a greater amount of synthesized geopolymer for MK1 and, consequently, a more compact structure with less interconnected pores. This fact was corroborated by the micrographs of both samples (Figures S1–S4) that showed a more compact structure for MK1. Thus, the higher conductivity in Stages IV–V, as well as the longer period in which Stage VI occurs for the MK1 sample, is probably correlated to a greater process of encapsulating MK particles after Stage III. Note that the σ_eff_ vs. t and dσ_eff_/dt curves illustrated a smoother reduction in electrical conductivity for the MK2 sample during Stage VI. This fact, correlated with the higher conductivity observed for the MK1 sample in Stage VII, may indicate the formation of a more compact structure for the MK1 sample with a smaller pore interconnection, making it difficult for the unbound water to be expelled and making it difficult to form microcracks, in addition to intensifying retraction processes.

## 4. Conclusions

In this study, the impedance spectroscopy technique was used to study the geopolymerization process of metakaolin for up to 7 curing days. This technique demonstrated excellent sensitivity in identifying changes in the geopolymerization process of different batches of metakaolin, to the detriment of subtle differences in their chemical composition or particle size. It was observed that the geopolymerization process in highly alkaline solutions could be divided into seven stages, including the processes of dissolution, nucleation, precipitation and formation of the gel and, eventually, the retraction/microcracks constitution. Late dissolution processes could be observed during the more advanced stages (up to Stage V) and were attributed to particles not being fully hydrated, which may originate, among other factors, from the rapid formation of hydrated products on the surface of some particles in the initial stages of the reaction, as observed in SEM images. The higher concentration of kaolinite verified by the TG/DTG, FTIR and XRD measurements for the MK2 sample was mainly demonstrated to have an influence on the initial stages (I–III) in the conductivity curves, mainly comprising the dissolution and amount of constituted geopolymer. The period in which Stage VI was observed was correlated to several mechanisms that act to increase the electrode contact resistance.

## Figures and Tables

**Table 1:** Chemical compositions (% in mass) of MK1 and MK2.

Oxides (%)	SiO_2_	Al_2_O_3_	Fe_2_O_3_	TiO_2_	K_2_O	Others	LOI
MK1	57.55	34.96	2.67	1.49	1.42	0.37	1.53
MK2	54.78	35.71	2.32	1.35	2.80	0.17	2.47

**Table 2:** Granulometric parameters (μm) obtained for MK1 and MK2.

	d(0.1)	d(0.5)	d(0.9)	Mean Particle Size
MK1	2.77	17.73	55.96	24.22
MK2	2.44	16.60	51.58	22.39

## Supplementary Materials

The following supporting information can be downloaded at: https://www.mdpi.com/article/10.3390/ma15238387/s1.
