# Supplementary material for: Impedance Spectroscopy as a Methodology to Evaluate the Reactivity of Metakaolin Based Geopolymers
Source: Materials (Basel). 2022 Nov 25;15(23):8387. doi: 10.3390/ma15238387 (PMC9740586; doi:10.3390/ma15238387)
Supplement: Supplementary file 1 [file materials-15-08387-s001.zip › materials-1996177-supplementary.pdf]

materials

MDPI

Supplementary material

Impedance Spectroscopy as a Methodology to Evaluate the Reactivity of Metakaolin Based Geopolymers

Danilo Bordan Istuque [1], Alex Otávio Sanches [1,*], Marcelo Bortoletto [1], José Antônio Malmonge [1], Lourdes Soriano [2], María Victoria Borrachero [2], Jordi Payá [2], Mauro M. Tashima [1,2,*] and Jorge Luis Akasaki [1]

1 MAC—Grupo de Pesquisa em Materiais Alternativos de Construção, Universidade Estadual Paulista (UNESP), Campus de Ilha Solteira, Av. Brasil Sul, 56-Centro, Ilha Solteira 15385-000, SP, Brazil

2 Institute of Concrete Science and Technology (ICITECH), Universitat Politècnica de València, 46022 València, Spain

* Correspondence: alex.o.sanches@unesp.br (A.O.S.); maumitta@upvnet.upv.es (M.M.T.)

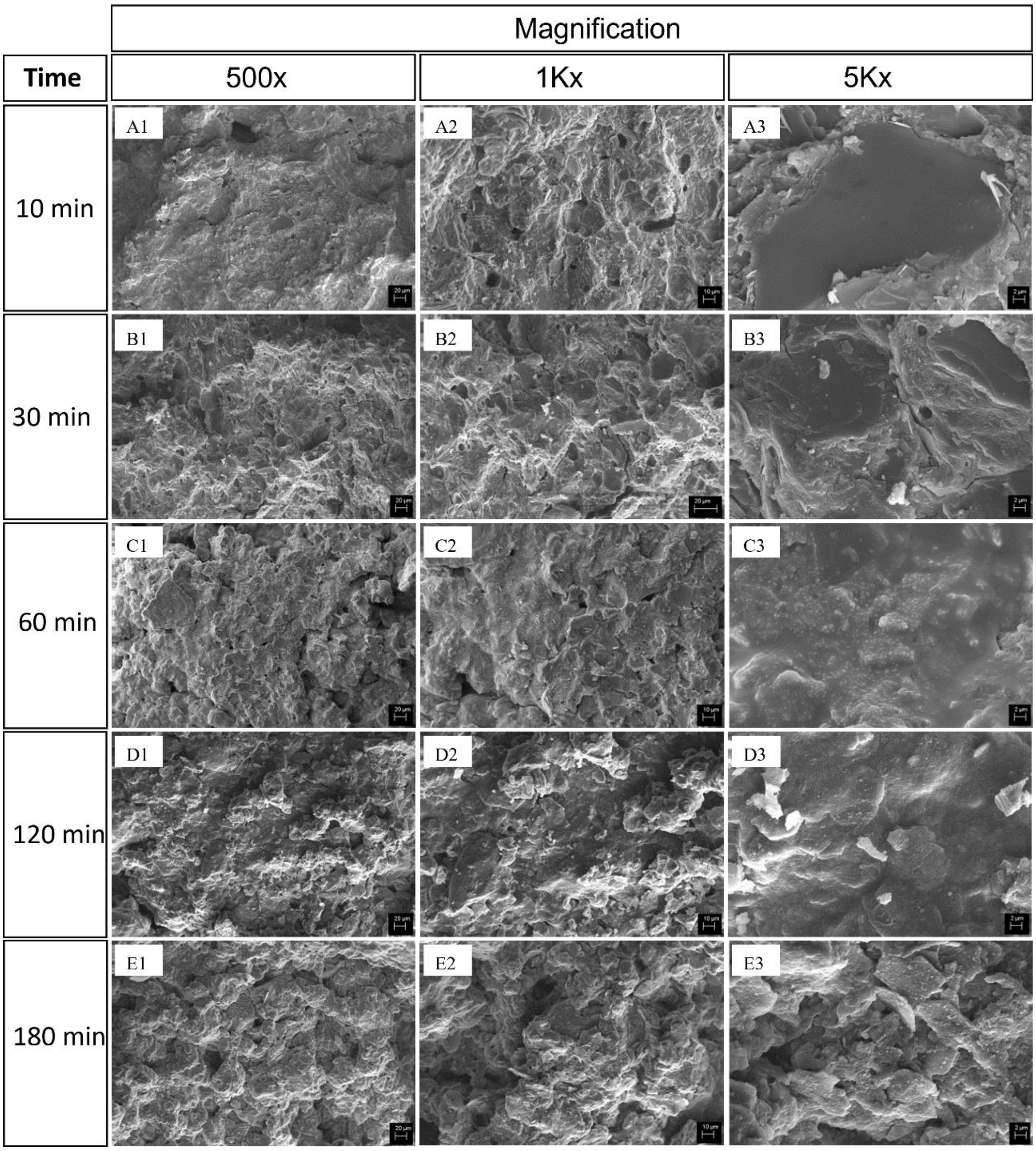

Figure S1 – SEM micrographs obtained from the fracture of the MK1 paste for different geopolymerization times (10-180 min).

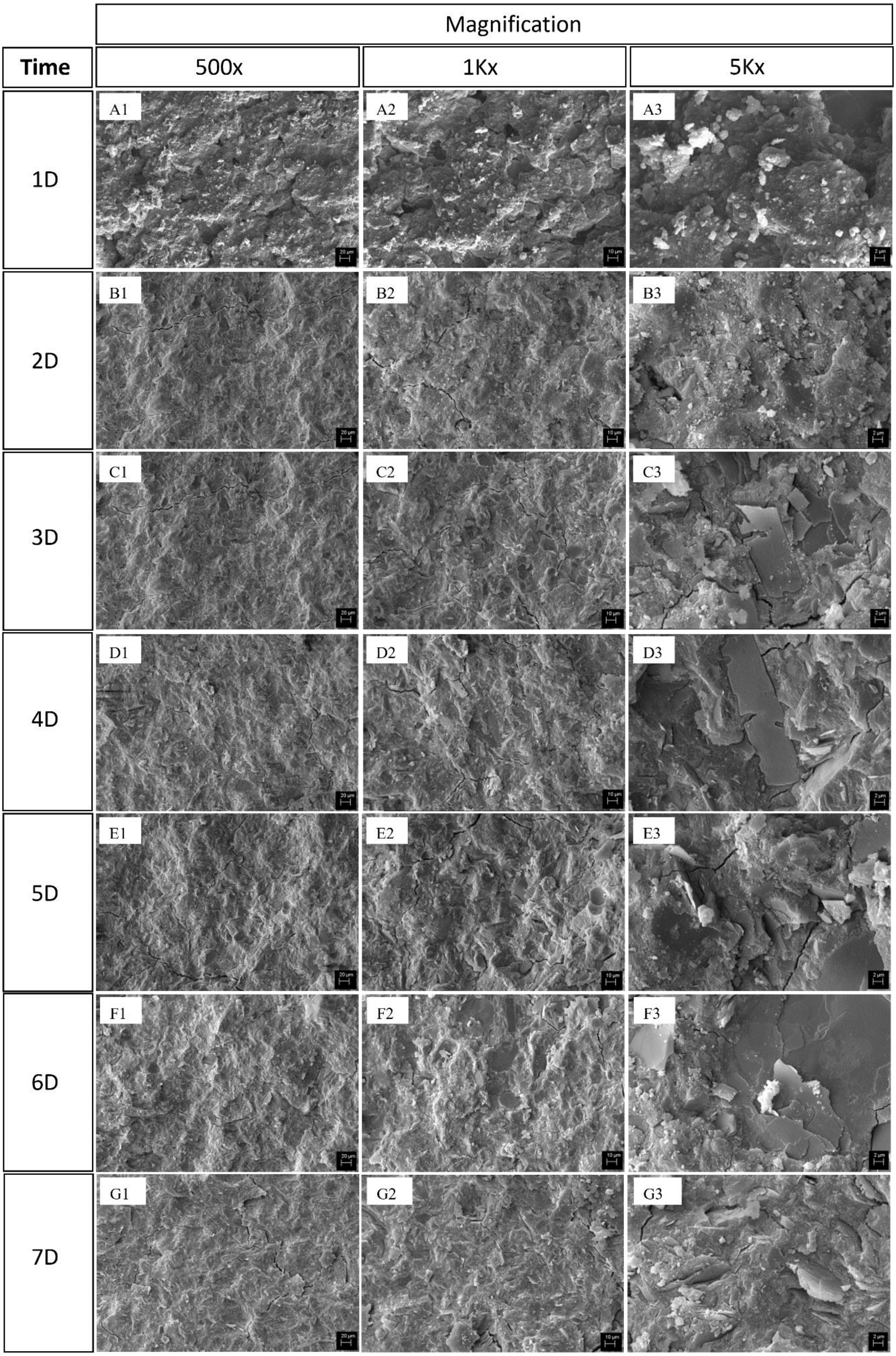

Figure S2. SEM micrographs obtained from the fracture of the MK1 paste for different geopolymerization times (1-7 days).

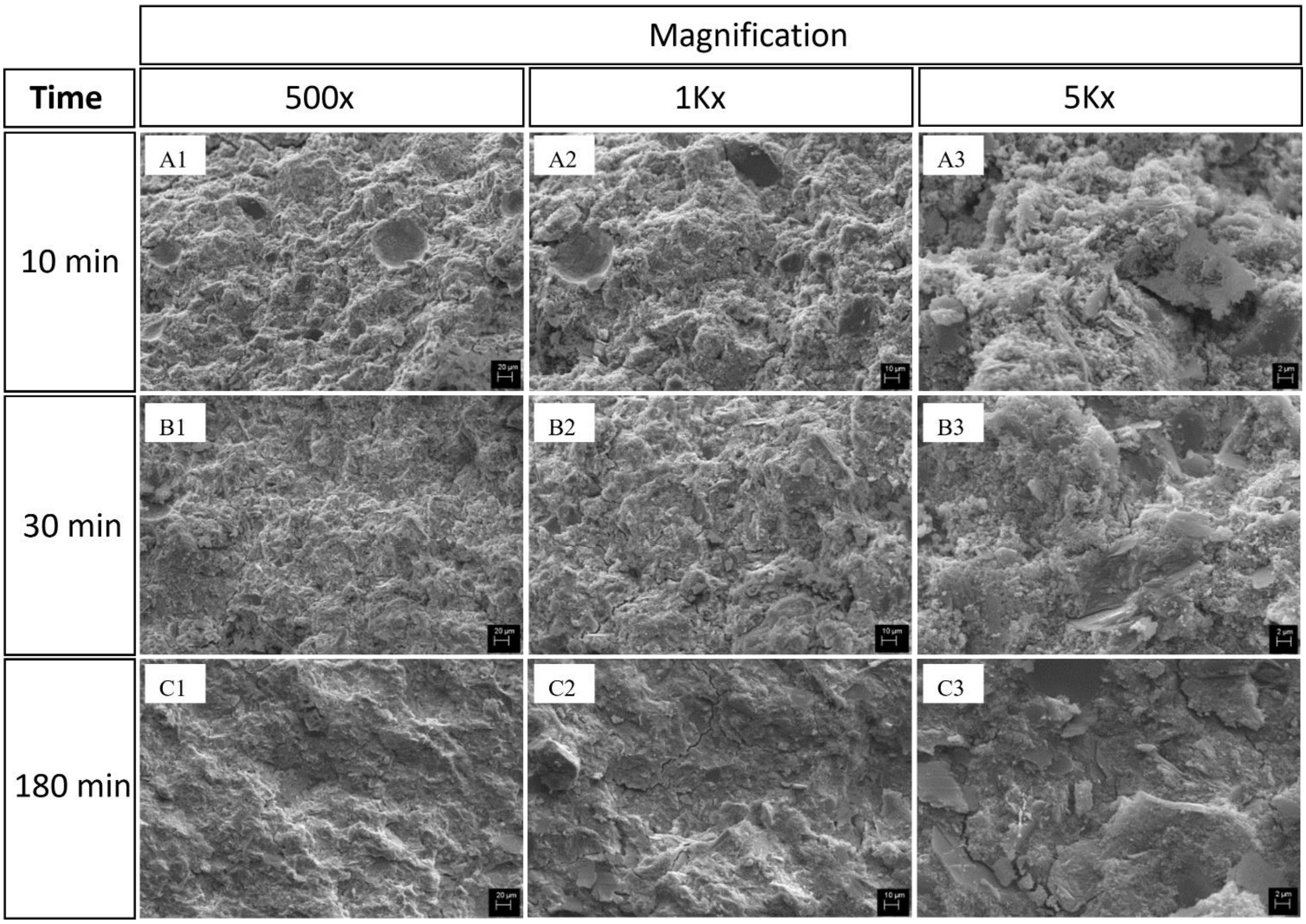

Figure S3. SEM obtained from the fracture of the MK2 paste for different geopolymerization periods(10-180 min).

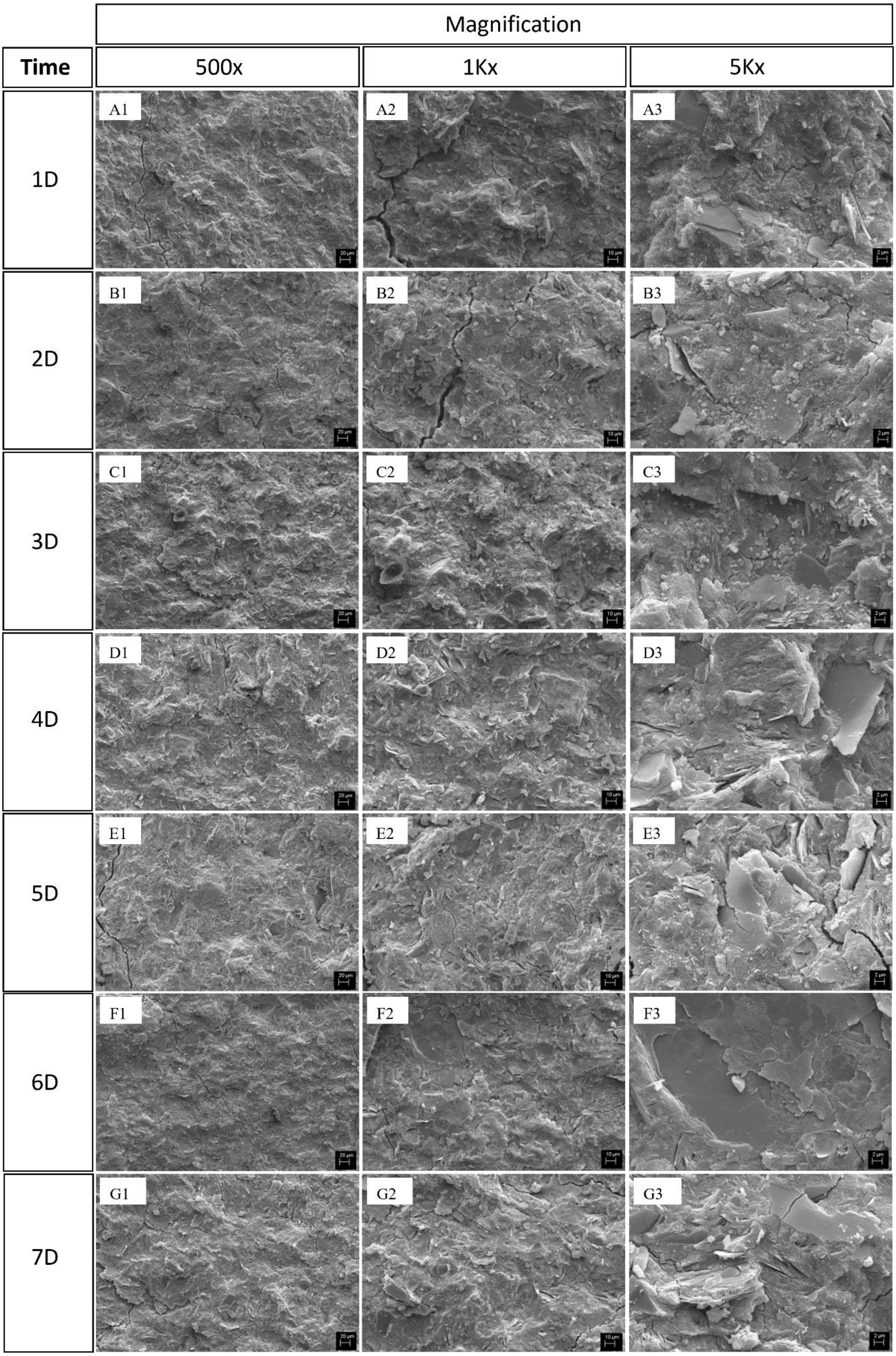

Figure S4. SEM obtained from the fracture of the MK2 paste for different geopolymerization periods (1-7days).
